# Where does the carbon go? A model–data intercomparison of vegetation carbon allocation and turnover processes at two temperate forest free-air CO_2_ enrichment sites

**DOI:** 10.1111/nph.12847

**Published:** 2014-05-21

**Authors:** Martin G De Kauwe, Belinda E Medlyn, Sönke Zaehle, Anthony P Walker, Michael C Dietze, Ying-Ping Wang, Yiqi Luo, Atul K Jain, Bassil El-Masri, Thomas Hickler, David Wårlind, Ensheng Weng, William J Parton, Peter E Thornton, Shusen Wang, I Colin Prentice, Shinichi Asao, Benjamin Smith, Heather R McCarthy, Colleen M Iversen, Paul J Hanson, Jeffrey M Warren, Ram Oren, Richard J Norby

**Affiliations:** 1 Department of Biological Sciences, Macquarie UniversitySydney, New South Wales, 2109, Australia; 2 Biogeochemical Integration Department, Max Planck Institute for BiogeochemistryHans-Knöll-Str. 10, 07745, Jena, Germany; 3 Environmental Sciences Division and Climate Change Science Institute, Oak Ridge National LaboratoryOak Ridge, TN, 37831-6301, USA; 4 Department of Earth and Environment, Boston UniversityBoston, MA, 02215, USA; 5 CSIRO Marine and Atmospheric Research and Centre for Australian Weather and Climate ResearchPrivate Bag #1, Aspendale, Victoria, 3195, Australia; 6 Department of Microbiology and Plant Biology, University of OklahomaNorman, OK, 73019, USA; 7 Department of Atmospheric Sciences, University of Illinois105 South Gregory Street, Urbana, IL, 61801, USA; 8 Biodiversity and Climate Research Centre (BiK-F) & Senckenberg Gesellschaft für NaturforschungSenckenberganlage 25, 60325, Frankfurt/Main, Germany; 9 Department of Physical Geography, Goethe-UniversityAltenhöferalle 1, 60438, Frankfurt/Main, Germany; 10 Department of Physical Geography and Ecosystem Science, Lund UniversityLund, Sweden; 11 Department of Ecology and Evolutionary Biology, Princeton UniversityPrinceton, NJ, 08544, USA; 12 Natural Resource Ecology Laboratory, Colorado State UniversityFort Collins, CO, 80523-1499, USA; 13 Canada Centre for Remote Sensing, Natural Resources CanadaOttawa, Canada; 14 AXA Chair of Biosphere and Climate Impacts, Department of Life Sciences, Grand Challenges in Ecosystems and the Environment and Grantham Institute for Climate Change, Imperial College LondonLondon, UK; 15 Department of Microbiology and Plant Biology, University of Oklahoma770 Van Vleet Oval, Norman, OK, 73019, USA; 16 Division of Environmental Science & Policy, Nicholas School of the Environment, Duke UniversityDurham, NC, 27708, USA; 17 Department of Forest Ecology & Management, Swedish University of Agricultural Sciences (SLU)SE-901 83, Umeå, Sweden

**Keywords:** allocation, carbon (C), climate change, CO_2_ fertilisation, elevated CO_2_, free-air CO_2_ enrichment (FACE), models, phenology

## Abstract

- Elevated atmospheric CO_2_ concentration (eCO_2_) has the potential to increase vegetation carbon storage if increased net primary production causes increased long-lived biomass. Model predictions of eCO_2_ effects on vegetation carbon storage depend on how allocation and turnover processes are represented.
- We used data from two temperate forest free-air CO_2_ enrichment (FACE) experiments to evaluate representations of allocation and turnover in 11 ecosystem models.
- Observed eCO_2_ effects on allocation were dynamic. Allocation schemes based on functional relationships among biomass fractions that vary with resource availability were best able to capture the general features of the observations. Allocation schemes based on constant fractions or resource limitations performed less well, with some models having unintended outcomes. Few models represent turnover processes mechanistically and there was wide variation in predictions of tissue lifespan. Consequently, models did not perform well at predicting eCO_2_ effects on vegetation carbon storage.
- Our recommendations to reduce uncertainty include: use of allocation schemes constrained by biomass fractions; careful testing of allocation schemes; and synthesis of allocation and turnover data in terms of model parameters. Data from intensively studied ecosystem manipulation experiments are invaluable for constraining models and we recommend that such experiments should attempt to fully quantify carbon, water and nutrient budgets.

## Introduction

Since the industrial revolution, burning fossil fuels and land-use change have driven an increase of *c*. 44% in the atmospheric concentration of carbon dioxide ([CO_2_]) (Le Quéré *et al.*, 2013). Current projections from coupled climate–carbon models suggest that the concentration may reach anywhere between *c*. 490 and 1370 ppm by 2100 (Moss *et al.*, 2010). Elevated [CO_2_] (eCO_2_) stimulates plant photosynthesis, which has the potential to increase net primary productivity (NPP) of vegetation (Kimball, 1983; Norby *et al.*, 2005). Many studies have investigated this NPP response, both experimentally using large-scale CO_2_ enrichment facilities, and also with ecosystem models (Oren *et al.*, 2001; Luo *et al.*, 2004; McCarthy *et al.*, 2010; Norby *et al.*, 2010; Drake *et al.*, 2011; Reich & Hobbie, 2012; Zaehle *et al.*, 2014).

Ultimately, however, the effect of eCO_2_ on NPP by itself is not as important as its consequences for key ecosystem properties, such as leaf area index (LAI) and vegetation carbon (C) storage. LAI is an important ecosystem property, with consequences for surface temperature and water balance. Vegetation C storage is a major component of the C cycle; *c*. 360 Pg C, or *c*. 20% of all terrestrial C, is stored in live forest biomass (Bonan, 2008; Pan *et al.*, 2011). Rising NPP due to CO_2_ fertilisation may lead to increased biomass C storage, which creates a strong negative feedback on rising atmospheric [CO_2_] (Canadell *et al.*, 2007; Le Quéré *et al.*, 2009). Increased NPP can also lead to increased input of plant detritus into the soil system, potentially increasing C storage in long-lived soil pools (Iversen *et al.*, 2012).

In order to predict changes in these ecosystem properties, we need to understand not only how eCO_2_ affects NPP, but also how it affects the allocation of the assimilated C to plant tissues. The effects of eCO_2_ on plant C storage will differ considerably if the C is allocated towards long-lived plant tissue (i.e. woody components), where it remains sequestered over long time periods; or alternatively, if cycling of C through the system is increased via increased allocation to short-lived tissues or reduced tissue lifespan (Luo *et al.*, 2003; Korner *et al.*, 2005). Similarly, the effects of eCO_2_ on LAI depend on changes in NPP but also on changes in the fraction of C allocated to foliage vs other plant components.

Currently, global vegetation models predict that eCO_2_ will lead to increasing C sequestration in both the biomass and soil (Cox *et al.*, 2000; Cramer *et al.*, 2001; Friedlingstein *et al.*, 2006; Lenton *et al.*, 2006; Schaphoff *et al.*, 2006; Thornton *et al.*, 2007; Arora *et al.*, 2013), but the simulated C-store (live biomass and soils) diverges considerably between simulations. Jones *et al.* (2013) showed a large spread in the simulated change in the land C-store of between *c*. −250 and 400 Pg C by 2100 from a series of model simulations run as part of the Coupled Model Intercomparison Project (CMIP5). There are many possible causes for this among-model variability, but one important difference among models is the representation of C allocation and pool turnover patterns. The choice of model allocation scheme has been shown to have significant consequences for predicted biomass responses. For example, Friedlingstein *et al.* (1999) showed that the CASA model would predict a 10% reduction in global biomass by replacing fixed empirical constants with a dynamic C allocation scheme based on resource availability (light, water and nitrogen (N)). Similarly, Ise *et al.* (2010) found large variability (up to 29%) among model estimates of woody biomass caused by different assumptions about C allocation coefficients. Weng & Luo (2011) evaluated the TECO model at the Duke site and found that partitioning to woody biomass to be the most sensitive parameter governing predictions of ecosystem carbon storage. Most recently, Friend *et al.* (2014) attributed uncertainty in multi-model predictions of the future vegetation store to different residence times in models.

In order to understand why models differ in their predictions of C sequestration, and to reduce this uncertainty, we need to identify the assumptions made in different models and examine how these assumptions impact on model predictions. Experimental data can then be used to help distinguish the best model assumptions. We applied a series of 11 ecosystem models to data from two temperate forest free-air CO_2_ enrichment (FACE) sites. In previous papers we used this assumption-centred modelling approach to examine model assumptions related to NPP and water use (De Kauwe *et al.*, 2013; Zaehle *et al.*, 2014; Walker *et al.*, 2014). In this paper, we focus on the processes of allocation and turnover. We document how each of the 11 models represent these processes. We then quantify how these process representations affect predictions of vegetation C storage and LAI, and compare the models against measurements at the two sites in order to understand which process representations have the capacity to capture observed responses.

In the absence of a mechanistic understanding of the processes controlling C allocation at the whole-plant level, models either follow empirical or evolutionary-based approaches (Franklin *et al.*, 2012). Empirical approaches include fixed coefficients, allometric scaling or functional balance approaches, while evolutionary-based approaches include optimisation, game-theoretic approaches and adaptive dynamics (Dybzinski *et al.*, 2011; Franklin *et al.*, 2012; Farrior *et al.*, 2013). The set of models used in this model intercomparison employed all of these approaches, with the exception of game theory and adaptive dynamics, which have not yet been widely employed in ecosystem models. We were therefore able to probe differences in the predicted CO_2_ responses of allocation processes among the most commonly employed model approaches.

## Materials and Methods

### Terminology

The terminology used to describe C allocation processes within the literature is rather ambiguous. Litton *et al.* (2007) proposed a series of definitions to standardise usage in experimental studies. Unfortunately, these definitions do not correspond directly to the way that processes are represented within most ecosystem models, which typically consider C allocation in terms of available NPP rather than Gross Primary Production (GPP). In this paper, therefore, we use terms that are defined according to typical ecosystem model structure. Many ecosystem models are based around differential equations for biomass, which can be most simply expressed as:

1
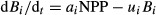
(*i*, ith plant component; *B*_*i*_, biomass of that component (kg m^−2^); *a*_*i*,_ fractions summing to 1; *u*_*i*_, turnover rates of each component (yr^−1^)). We considered the plant components to be foliage, wood (including stem, branch and coarse roots), fine roots and reproduction. We defined ‘allocation coefficients’ to mean the fractions *a*_*i*_ that determine the division of NPP among the plant components. We also defined ‘biomass fractions’ to mean the fraction of total plant biomass present in each component at a given time. As can be seen from Eqn (1), the biomass fractions depend both on the allocation coefficients and turnover rates.

### Experimental data

Models were applied to two experimental sites, both of which have been extensively described elsewhere (Norby *et al.*, 2001; McCarthy *et al.*, 2010; Walker *et al.*, 2014). The Duke FACE site was situated in a loblolly pine (*Pinus taeda*) plantation in North Carolina, USA (35.97°N, 79.08°W). The Duke experiment was initiated in 1996, when trees were 13 yr old. By the end of the experiment (2007), there was a significant hardwood understorey in addition to the overstorey pines. Data used in this paper refer to the forest stand as a whole, thus including both pines and hardwoods, because fine root production data were not separated by species. Six 30-m diameter plots were established, and CO_2_ treatments were initiated in August 1996. Three of these plots tracked ambient conditions and three plots received continuous enhanced CO_2_ concentrations of +200 μmol mol^−1^ (mean *c*. 542 μmol mol^−1^).

The ORNL FACE site was located in Tennessee, USA, at the Oak Ridge National Laboratory (35.9°N, 84.33°W) and is a sweetgum (*Liquidambar styraciflua*) plantation, established in 1988 on a former grassland. Treatment began at Oak Ridge in 1998 with two elevated rings (*c*. 25 m diameter) with an average growing season [CO_2_] of 547 μmol mol^−1^ and three ambient CO_2_ (aCO_2_) rings (*c*. 395 μmol mol^−1^).

Detailed measurements were collected during the experiments at both sites. Data used in this study included biomass, litterfall and NPP of each component (foliage, wood and fine root), and total leaf area index (LAI). NPP at both sites was calculated as the sum of woody biomass increment (estimated from allometric relationships between biomass and tree diameter and height), foliage productions (from litter traps), and fine-root production (from minirhizotron observations), as fully described by Norby *et al.* (2005) and references cited therein. At Duke FACE, observations of growth and litter components were only available from 1996 to 2005, whereas at ORNL FACE observations were available from 1998 to 2008. In this study we analysed model results for the corresponding periods for which we had observations, that is, 1996–2005 at Duke and 1998–2008 at Oak Ridge. These data are described in detail elsewhere, for Duke in McCarthy *et al.* (2007, 2010) and for Oak Ridge in Norby *et al.* (2001, 2004), and Iversen *et al.* (2008). These datasets are available at: http://public.ornl.gov/face/index.shtml.

From these data we calculated annual allocation coefficients for the foliage, wood, fine roots (growth of coarse roots was included in the wood component) and reproduction over the whole experiment. Allocation coefficients were calculated as NPP of individual components divided by total NPP. Turnover coefficients were calculated on an annual basis as the annual sum of litter divided by the annual maximum of each biomass component (foliage, wood and fine roots). The lifespan of each component is defined as the inverse of the turnover coefficients. In addition, we calculated whole-canopy specific leaf area as LAI divided by foliage biomass.

### Model simulations

The 11 models applied to the two FACE sites include stand (GDAY, CENTURY, TECO), age/size-gap (ED2, LPJ-GUESS), land surface (CABLE, CLM4, EALCO, ISAM, O-CN) and dynamic vegetation models (SDGVM). A detailed overview of the models is given in Walker *et al.* (2014), and detailed analyses of the water and N cycle responses are provided by De Kauwe *et al.* (2013) and Zaehle *et al.* (2014) respectively.

Each model was used to run simulations covering 1996–2008 at the Duke FACE site and 1998–2009 at the ORNL FACE site. Modellers were provided with general site characteristics, meteorological forcing and CO_2_ concentration data. Most models simulated the Duke FACE site as a coniferous evergreen canopy, although ED2 and LPJ-GUESS included a hardwood fraction. All models simulated the ORNL FACE site as a broadleaf deciduous canopy (Walker *et al.*, 2014). Models output a variety of C, N and water fluxes at their appropriate driving resolution (hourly or daily).

### Analysis approach

We deliberately did not statistically evaluate any of the models against observations, because models can easily yield quantitatively good responses for incorrect reasons; thus such an approach typically does not correctly diagnose model deficiencies (Medlyn *et al.*, 2005; Abramowitz *et al.*, 2008; Walker *et al.*, 2014). Furthermore, at both sites a series of storm events introduced transient system responses which are not accounted for in the models, complicating direct point comparisons. Instead, we assessed the model performance qualitatively by attempting to understand the predictions made based on the underlying assumptions relating to allocation and turnover processes. In assessing model performance, a ‘good’ model is one that captures the processes underlying response of the system to eCO_2_, although it may not explicitly match the temporal dynamics observed at individual sites.

We first documented how the allocation process is represented in each of the 11 models. For some models, the sum of annual plant growth does not exactly equal total photosynthesis less respiration in each year, due to the presence of a nonstructural labile carbon pool. The modelled size of this pool varies among models depending on how transfer from storage to growth is represented, but within a model remains relatively constant over the course of the experiment (see Supporting Information Notes S1, Fig. S1a,b). Because there are no estimates of this pool size for either experiment, we could not evaluate the modelled labile C pool against data. In what follows, therefore, we focus on the allocation of carbon used for growth among different plant tissues. We calculated the allocation coefficients *a*_*i*_ from model outputs of annual growth of each plant component, and compared the modelled allocation coefficients at aCO_2_ and elevated CO_2_ (eCO_2_) at the two sites against the observed values. The results for the allocation coefficients were interpreted in terms of the underlying model representation of allocation.

We then examined the models' predicted CO_2_ responses of leaf area index (LAI, representing canopy cover) and C sequestration in woody biomass. Predicted LAI depends on specific leaf area (SLA), the ratio of leaf area to leaf mass, as well as allocation coefficients. Therefore, we also documented how the models represented SLA. Similarly, predicted C sequestration also depends on tissue turnover, so we documented how the models represented turnover. Finally, we analysed how the representations of these processes combined to determine the model predictions.

### Model representations of allocation

We classified the ways that C allocation is implemented in the models into four general classes: (1) fixed coefficients; (2) functional relationships; (3) resource limitations; and (4) optimisation. In *fixed-coefficient* models, a fixed fraction of NPP is allocated to each plant component. In *functional-relationship* models, relationships among plant organs provide constraints from which the allocation coefficients can be determined. In general, these relationships are based on the hypotheses that (1) sapwood cross-sectional area must be sufficient to supply structural support and water transport for the leaf area (the pipe-model hypothesis, Shinozaki *et al.*, 1964a,b) and (2) root activity and leaf activity should be balanced (the functional balance hypothesis, Davidson, 1969). In *resource-limitation* models, the allocation coefficients are adjusted according to which resource is most limiting to growth. Resource-limitation models are based on similar ideas to the functional relationship models, but the key distinction is that relationships are calculated among allocation coefficients rather than among the biomass fractions. In *optimisation* models, allocation coefficients are varied to maximise some measure of performance by the plants.

Each of the 11 ecosystem models was classified into one of these four groups. Classifications and a full description of how each model represents allocation are given in Table 1. Many models separately consider allocation to bole, branches and coarse roots, whereas others lump these components into wood. Here, we consider only the combined component wood to enable comparison among models. Three models, ED2, LPJ-GUESS and O-CN, also utilise a proportion of available C for reproduction.

**Table 1:** Full description of the assumptions regarding allocation made in the models for the simulations in this paper

Model	Representation of allocation	Timestep
Fixed coefficients		
CABLE	Allocation coefficients are fixed, but fractions differ between three phenological phases: (1) maximal leaf growth phase: 80% of available C allocated to foliage; 10% each to wood and roots (2) steady growth phase: plant functional type (PFT)- specific allocation coefficients used (3) final phase: no leaf growth; available C allocated to wood and roots in ratio 55%: 45%	Daily
CLM4	For this study, allocation fractions were set as fixed empirical constants based on site observations, which did not vary through the year. Note: The standard version of the model allocates C to the stem and foliage as a dynamic function of NPP.	Daily
EALCO	For this study, allocation coefficients were determined to maintain a prescribed relationship among plant tissues, namely: foliage: sap wood: fine root = 1: 0.75: 0.5 for conifers and = 1: 3: 2 for deciduous trees The start of plant growth is determined by a temperature sum. During the early growing season, all available C is allocated to foliage because leaf biomass is small relative to sapwood and fine roots. Leaves stop growing when LAI reaches a maximum LAI that is prescribed for each year and treatment based on the site data. After LAI reaches its maximum, available C is allocated to sapwood and fine root only to maintain their prescribed relationship mentioned above (i.e. 60% vs 40%). The growth of coarse roots and heartwood occurs during the senescence of fine root and sapwood, respectively On an annual basis, the outcome of this set of assumptions is that root vs sapwood allocation relationship is fixed, and foliage allocation yields the observed maximum LAI when enough C is fixed by the plants Note: in other work the model EALCO often uses a ‘transport resistance scheme’ where flows of C and N depend on concentration gradients (Thornley, 1972; Wang *et al.*, 2002)	Daily
GDAY	Allocation fractions are empirical constants set from site observations. Theses coefficients were varied between ambient and eCO_2_ treatments at ORNL to reflect empirical site measurements	Annual
Functional relationships		
ED2	Allocation is determined such that the biomass components follow allometric relationships given by Medvigy *et al.* (2009): 2 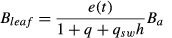 3 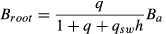 4 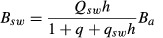 (*B*_*a*_, active biomass pool; *B*_*leaf*_, *B*_*root*_ and *B*_*wood,*_ biomass pools of foliage, sapwood and roots, respectively). Leaf phenology is described by a phenology parameter (e(t)) [0–1]). Sapwood biomass and peak leaf biomass are maintained in the proportion *q*_sw_ *h* (*h*, tree height; *q*_*sw*_, fixed leaf : sapwood area ratio). Root biomass and peak leaf biomass are maintained in the ratio *q*, which increases with increasing water or nitrogen limitation. After allocating to leaves and roots on a daily basis, ED2 uses a 70 : 30 split of available ‘reserve’ C between woody growth and reproduction Note: in the standard ED2 model, allocation fractions do not vary with N limitation	Daily
LPJ-GUESS	A new version of the model incorporating N limitation was used (Smith *et al.*, 2014). The allocation model follows Sitch *et al.* (2003), with the addition of N dependence of the leaf: root biomass ratio First, 10% of NPP is allocated to reproduction. The remaining NPP is allocated to the foliage, wood and roots on an annual time step based on allometric relationships among biomass components The ratio of LAI to sapwood area (*SA*) is constant 5 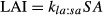 (*k*_*la:sa*_, a PFT-dependent constant). Additionally, upward tree growth requires an increase in supporting stem diameter 6 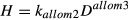 (*H*, tree height; *D*, stem diameter; *k*_*allom*2_ and *k*_*allom*3_, PFT dependent allometric constants). These two relationships define the wood biomass to leaf biomass ratio The root biomass to leaf biomass ratio depends on a PFT-specific maximum leaf-to-root mass ratio *lr*_*max*_ and N and water availability factors (*N* and *W*, ranging 0–1): 7 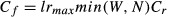 (*C*_*r*_, root biomass pool; *C*_f_, foliage biomass pool)	Annual
O-CN	Implements the same scheme as LPJ-GUESS, with the key changes being that: (1) allocation takes place on a daily time step, (2) the leaf-to-root mass ratio and leaf-to-sapwood ratios do not vary with PFT, and (3) partitioning of NPP to reproduction also occurs on a daily basis and depends on the amount of remaining NPP after allocation to foliage, wood and fine roots has taken place. A fast turnover labile pool buffers NPP against short-term variations in GPP; and a nonrespiring reserve pool buffers interannual variability and facilitates bud burst in deciduous trees	Daily
Resource limitations		
DAYCENT	Carbon is allocated according to priorities. Fine roots have first priority, then foliage and finally wood. Demand by the fine roots varies between 5% and 18% of total NPP depending on the maximum of two limitations (soil water and nutrient availability). The remaining carbon available for allocation is then distributed to the foliage pool until the maximum LAI is reached. The maximum LAI is set for each PFT depending on an allometric relationship with wood biomass. Allocation to woody tissue only takes place once the maximum LAI has been attained	Daily
ISAM	Allocation formulation after Arora & Boer (2005), with a dependence on light and water availability (but not explicitly nutrient limitation). Under high LAI, light limitation occurs, and allocation to wood increases to compete for light. When water limitation occurs, allocation to roots increases. Allocation to foliage is calculated as the residual. The allocation fractions are calculated as follows: 8 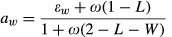 9 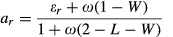 10 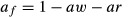 (*W*, soil water availability factor [0–1]; *L*, light availability factor; *ω ɛ*_*w*_,*ɛ*_*r*_, PFT-dependent allocation parameters). *L* is given by *L* = exp(–*k* LAI), (*k*, light extinction coefficient; LAI, leaf area index, which is input from observations). For broadleaf PFTs, this scheme is modified using three phenological growth phases: (1) Leaf onset phase: allocation is completely to leaves, with zero allocation to wood or roots (2) Steady growth phase: resource limitation model used (3) Leaf senescence phase: allocation to foliage is set to zero, and *a*_*w*_ and *a*_*r*_ are increased to sum to one The phases are determined by the ratio of LAI to a maximum LAI value for the biome. Phase (2) starts once the LAI reaches half the maximum LAI, and ends once LAI falls below 95% of the maximum LAI value	Daily
TECO	The total amount of carbon available for allocation on a given day is given by the tissue growth rate (*G*)*,* which is a function of temperature and water availability. The model prioritises allocation to foliage and roots. The demand for carbon by foliage is given by the amount of carbon needed to reach the maximum LAI. Growth is allocated to foliage to meet this demand, but at any time step the allocation cannot exceed 40% of the total available carbon to be exported. Demand for carbon by the roots increases with decreasing water availability, but cannot exceed 30% of the total available carbon to be exported. The remaining available carbon is then allocated to the stem. The allocation coefficients are thus calculated as follows: 11 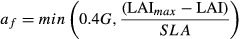 12 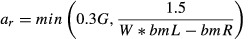 13 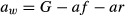 (*G*, total carbon to be allocated; LAI_*max*_, PFT-specific maximum leaf area index; SLA, specific leaf area; *W*, soil water availability factor [0–1]; bmL and bmR, parameters defining the ratio of fine roots to foliage). LAI_*max*_ depends on canopy height, but height was assumed constant in these simulations for both PFTs. The maximum LAI thus did not vary in TECO, unlike the other models	Daily
Optimisation		
SDGVM	SDGVM optimises canopy LAI such that net canopy C uptake is maximised. The annual carbon balance of the lowest canopy layer is calculated. Allocation to foliage in the current year is determined such that the lowest layer of the canopy had a positive carbon balance in the previous year. Allocation of remaining labile carbon between roots and woody tissue are given by constant PFT-specific fractions	Daily

Note that in several instances, alternative allocation sub-models are available for the models used here, so other applications of these models may not use the allocation routines described here.

## Results

### Allocation patterns

**Fig 1:**
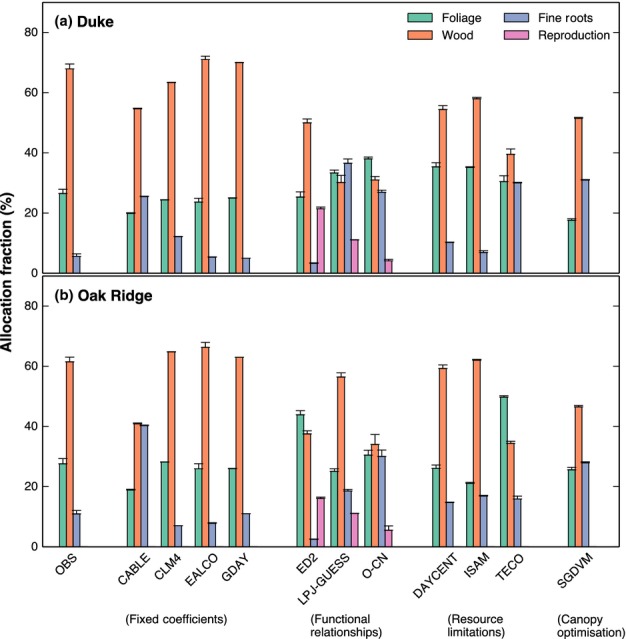
Fractions of Net Primary Productivity (NPP) allocated at ambient CO_2_ to the foliage, wood, fine roots and reproduction at (a) Duke and (b) Oak Ridge. The values shown are means of the annual values and the error bars show the interannual variability in allocation fractions (± 1SD) calculated over the number of years (*n*) of the experiment (*n* = 10 at Duke and *n* = 11 at Oak Ridge). Models are grouped by allocation model type. Observations are shown by the abbreviation ‘OBS’. Further discussion of differences among model predictions of allocation patterns at ambient CO_2_ concentration is provided in Table 1 and in Supporting Information Notes S2.

Figure 1 shows the average measured and modelled C allocation coefficients in the ambient treatments over the experimental period at both sites. At both sites, the observations indicate that the largest fraction of NPP goes to wood, but at Duke the wood allocation fraction is greater, and the root allocation fraction lower, than at ORNL. Overall, the models agree with the observations that the greatest fraction of NPP was allocated to woody tissue at both sites, with notable exceptions being LPJ-GUESS and O-CN at Duke, and O-CN and TECO at ORNL. Most differences among models in their prediction of allocation fractions at ambient CO_2_ arise from parameterisation; these differences are discussed in the Notes S2.

The data indicate that eCO_2_ had very different effects on allocation patterns at the two sites (Figs3). At Oak Ridge, trees in eCO_2_ increased allocation towards fine-root production at the expense of wood and leaves. As a consequence, root production roughly doubled at soil depths below 0.3 m (Iversen *et al.*, 2008). By contrast, at Duke, the root biomass proportion also increased at depth (Pritchard *et al.*, 2008), but the root allocation fraction did not change. There was a shift instead from foliage allocation to wood allocation, with the average wood allocation fraction increasing by 3%, although this shift was not statistically significant (95% CI = −1.4%, 7.4%) (McCarthy *et al.*, 2010).

**Fig 2:**
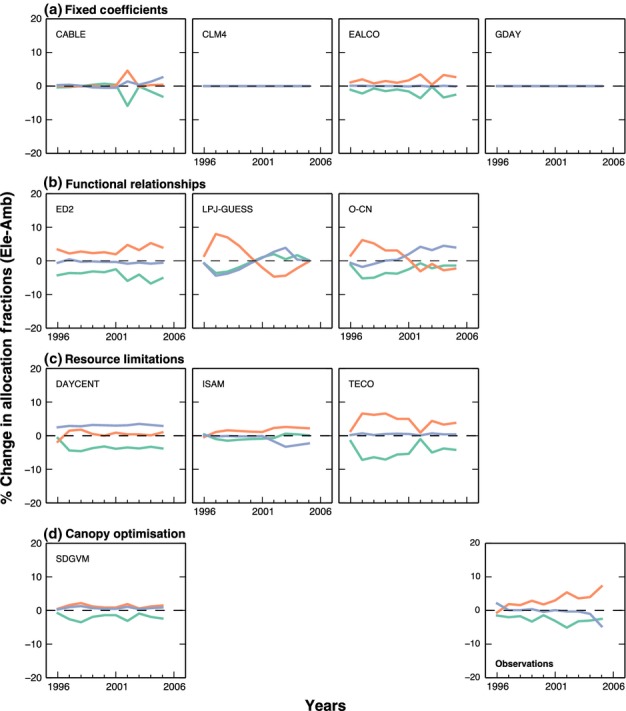
Change in the percentage of annual Net Primary Productivity (NPP) allocated to the foliage (green line), wood (orange line) and fine roots (blue line) between ambient and elevated CO_2_ at Duke.

**Fig 3:**
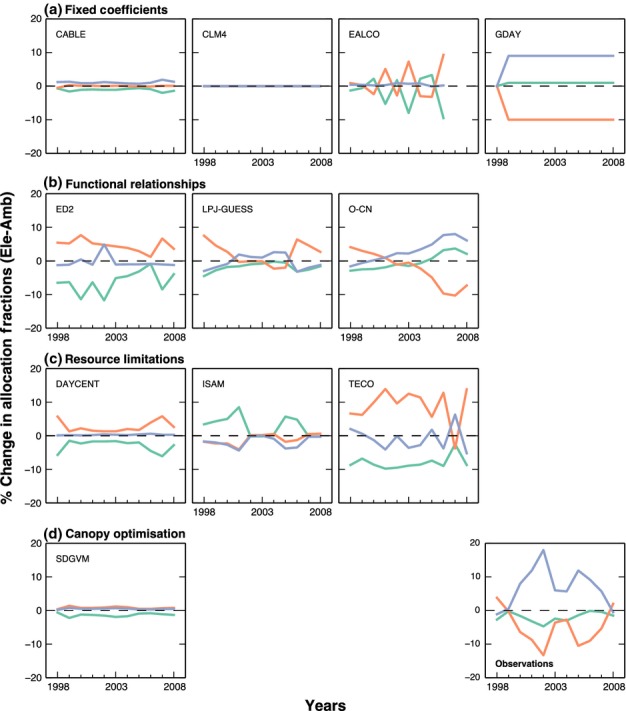
Change in the percentage of annual Net Primary Productivity (NPP) allocated to the foliage (green line), wood (orange line) and fine roots (blue line) between ambient and elevated CO_2_ at Oak Ridge.

In general, the models predicted a reduction in foliage allocation in response to CO_2_ but disagreed on where the additional NPP would be partitioned (Figs3). Differences among models at ambient and in response to eCO_2_ can be understood following the categorisation of allocation schemes described in the Materials and Methods section.

#### Fixed coefficients

Fixed coefficient models assume that allocation fractions are not affected by environmental conditions. In two of these models, CLM4 and GDAY (at Duke), there was no change in allocation in response to eCO_2_ (Figs2a, 3a). At Oak Ridge, GDAY assumed that root allocation was increased in response to eCO_2_, based on the average CO_2_ response measured at the site. It can be seen in Fig. 3 that this response is assumed to start in the second year of the experiment, because in the deciduous version of the model, growth is based on the previous year's accumulated productivity. These models are included for completeness but overall, the observations from both experiments indicate that allocation responses to eCO_2_ are dynamic, so it is clear that the constant coefficient approach is of limited usefulness for predicting allocation patterns under eCO_2_.

Somewhat surprisingly, two other fixed coefficient models, CABLE and EALCO, did show eCO_2_ effects on allocation (Figs2a, 3a). These effects occur because both models use phenological phases, with different fixed allocation coefficients during each phase (Table 1). As a result, eCO_2_ can alter annual allocation coefficients, even though the allocation coefficients are fixed during each growth phase, because the relative CO_2_ enhancement of NPP varies throughout the year. For CABLE at Duke, this effect is clearly seen during the drought year (2002) in Fig. 2. The drought occured after foliage expansion, during a period when allocation to foliage is low and allocation to wood is high. The CO_2_ effect on NPP during drought is amplified. Thus, the CO_2_ effect is largest during the period when wood allocation is greatest, with the overall effect that allocation to wood increases at the expense of foliage. Although such a drought × CO_2_ interaction on allocation is also predicted by other types of allocation models (e.g. see ED and SDGVM, Fig. 2), in this model it was not intentional, but rather was a side effect of the assumption of phenological phases for allocation.

In EALCO, the assumption that the period of foliage allocation continues until the observed maximum LAI is reached implies that annual foliage allocation is determined by the observed LAI. The fine-root allocation coefficient is fixed, and wood allocation is therefore the remainder of NPP. At Duke, where observed root allocation was not affected by eCO_2_, the allocation patterns simulated by EALCO resemble the observations (Fig. 2). At Oak Ridge, by contrast, where observed root allocation was strongly affected by eCO_2_, the allocation patterns simulated by EALCO differ strongly from the observations (Fig. 3). As with CABLE, however, these eCO_2_ effects were an unintended consequence of the phenology of the allocation scheme.

#### Functional relationships

The three models ED2, LPJ-GUESS and O-CN allocate C according to functional relationships among plant organs, which maintain sapwood, foliage and fine roots in ratios that vary according to N and water availability. The allocation responses to CO_2_ predicted by these three models are relatively consistent (Figs2b, 3b), and capture the observed responses to some extent. With the additional increase in productivity in response to CO_2_, all three models predict that initially wood allocation must increase to supply the extra wood volume necessary to maintain the same leaf to sapwood area ratio. In ED2, this effect continues throughout the experiment, because high available soil N means that nutrient limitation does not develop (Zaehle *et al.*, 2014). In LPJ-GUESS and O-CN, water and nutrient limitations develop over the course of the experiment, causing allocation to shift towards roots to maintain a functional balance between foliage and roots. This effect is seen most clearly in O-CN, in which increased N stress develops at both sites. In LPJ-GUESS, the dynamics of allocation at Oak Ridge change following a simulated mortality event in 2005. The mortality reduces the stand-scale leaf:sapwood area ratio significantly, driving an increase in wood allocation in the last years of the experiment.

#### Resource limitations

Three models, ISAM, DAYCENT and TECO use resource limitation approaches, in which allocation coefficients are determined by limitations of water, light and nutrient availability. Although the approaches are similar in theory, the implementations are sufficiently different that the three models predict rather different allocation patterns and responses to eCO_2_ (Figs3).

In ISAM, the allocation coefficients vary with water and light limitation (Table 1). However, the predicted CO_2_ effects on allocation differ between the sites because of the use of phenological phases in deciduous species. At Duke, eCO_2_ increased LAI, decreasing light availability, and reduced transpiration per unit leaf area, increasing water availability. Both effects cause an increase in wood allocation (Fig. 2g), much like that predicted by the allometric models, and somewhat similar to observations. By contrast, at Oak Ridge, foliage allocation is predicted to increase strongly with eCO_2_ (Fig. 3g), as an unintentional side effect of the use of phenological phases. The start of senescence period (the third phenological phase) occurs when the observed LAI declines to 95% of the prescribed maximum value. Because LAI is greater in the eCO_2_ treatment, the LAI does not fall below the senescence threshold until considerably later than in the ambient treatment (*c*. 20 d). As allocation to foliage continues until the senescence phase starts, foliage allocation is increased considerably in response to CO_2_, in stark contrast to observations and other models.

The DAYCENT and TECO models use similar prioritisation schemes to decide allocation (Table 1). However, the predicted response of allocation to eCO_2_ differs between these two models because of different predicted impacts on water and nutrient stress. In DAYCENT, at Duke, root allocation was increased with eCO_2_ due to an increase in nutrient limitation. At Oak Ridge, by contrast, root allocation was unchanged, indicating that water and nutrient stress were unaffected by eCO_2_. At both sites, foliage allocation decreased in response to eCO_2_ because the maximum prescribed LAI had been attained. As a result, allocation to wood (the third in the list of priorities) increased at Oak Ridge, but not at Duke. These predictions differed markedly from observations at both sites.

In the TECO model, at Duke, the maximum root allocation was obtained at aCO_2_ and as result there was no CO_2_-induced change. At Oak Ridge, water stress was reduced under eCO_2_ as a consequence of water savings due to stomatal closure, resulting in lower root allocation. At both sites, foliage allocation was reduced as LAI approached the prescribed maxima, as in DAYCENT. Consequently, according to the prioritisation scheme, allocation to wood is increased at both sites, and most strongly at Oak Ridge. These predictions are similar to observed allocation responses at Duke, but very different from observations at Oak Ridge.

#### Canopy optimisation

In SDGVM, LAI is varied to maximise net canopy C uptake (photosynthesis less respiration and leaf C costs). This optimisation determines the amount of C allocated to foliage; the rest of the C available is allocated to wood and roots in a fixed ratio. This approach predicts that allocation to foliage should decrease at both sites (Figs2d, 3d) because the eCO_2_ enhancement in NPP is greater than the LAI increase predicted by the optimisation scheme. The changes in foliage allocation predicted by this model are similar to observations. However, because the model assumes that the remaining NPP is divided in a fixed fraction between wood and roots, it did not successfully predict changes in wood and root allocation.

### Consequences for Leaf Area Index

**Fig 4:**
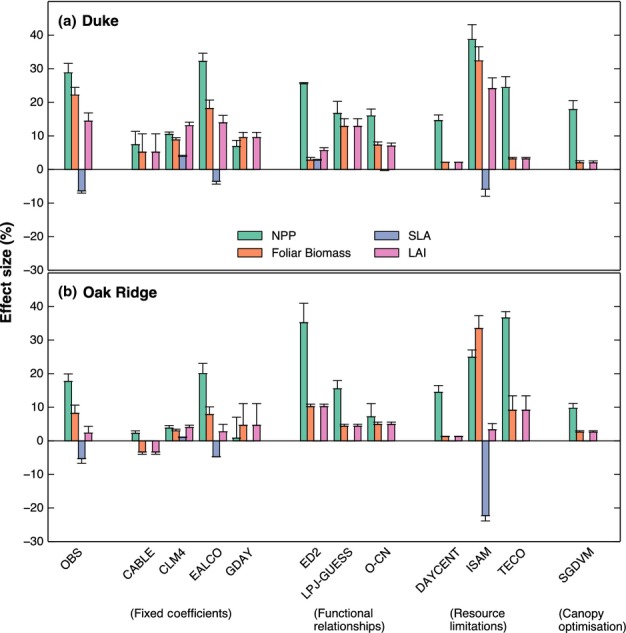
Response (elevated/ambient) of Net Primary Productivity (NPP), foliar biomass, whole-canopy specific leaf area (SLA) and leaf area index (LAI) to CO_2_ enhancement at Duke (a) and Oak Ridge (b). The data shown are means over the years of the experimental measurements (Duke, 1996–2005; Oak Ridge, 1998–2008), with error bars indicating interannual variability (± 1 SD). Foliage biomass and LAI data are means of the maximum value simulated/observed during each year. SLA is calculated as whole-canopy LAI divided by foliage biomass. Observations are shown by the abbreviation ‘OBS’.

Differences in model predictions of ambient LAI are discussed in Walker *et al.* (2014); here we focus on the predicted eCO_2_ effect on LAI. This effect depends, first, on the NPP enhancement; second, on the change in allocation of NPP to foliage; and, third, on any change in specific leaf area (SLA) with eCO_2_. Fig. 4 shows the observed and modelled responses of NPP, foliar biomass, SLA and LAI to eCO_2_. Most models predict that eCO_2_ leads to an increase in NPP, but there is a reduction in foliage allocation, such that the increase in foliage biomass is less than the increase in NPP. These predictions are generally consistent with the observations. The exception to this rule is ISAM at Oak Ridge, where foliage allocation increased, as explained above, leading to a larger response of foliage biomass than of NPP.

Observations from both sites showed that whole-canopy SLA (calculated as total leaf area index divided by total leaf biomass) was reduced at eCO_2_ (−6.4 and −5.3% at Duke and Oak Ridge, respectively). Owing to this reduction in SLA, the observations show smaller CO_2_ effects on LAI (14.4% and 2.3% increase at Duke and Oak Ridge, respectively) compared to the effects on foliage biomass (22.1% and 8.2% increase at Duke and Oak Ridge, respectively). By contrast, most models assume that SLA is constant, and therefore the enhancement in LAI due to CO_2_ directly corresponds to the foliage biomass enhancement.

However, some models vary SLA. In CLM4, SLA increases as a linear function of canopy depth (Thornton & Zimmermann, 2007). Increased foliage allocation under eCO_2_ increases LAI, which results in a lower mean foliage C cost (increased mean SLA), allowing the enhancement in LAI to be greater than the corresponding foliar biomass enhancement. This response of SLA is in the opposite direction to observations; data at both sites indicate a reduction in SLA at eCO_2_. In the ISAM model, LAI is decoupled from canopy biomass. The LAI is calculated based on a phenological model where the maximum LAI is specified, and has no relationship with the foliage biomass. As a consequence, the implied SLA can change dramatically with eCO_2_, as at Oak Ridge where foliage biomass is predicted to increase considerably but prescribed LAI does not (Fig. 4). In the EALCO model, SLA is forced to decrease at eCO_2_ by a percentage that is based on observations. By including this observation into the model procedure, the EALCO model is able to replicate the CO_2_ effects on both foliage biomass and LAI (Fig. 4).

### Biomass turnover

The eCO_2_ effect on biomass C storage depends both on allocation patterns and turnover times. We therefore documented the turnover times for different plant tissues in both observations and models (Tables3). In comparing model turnover times to observations, it is important to bear in mind that observed turnover times are calculated from the turnover and mortality of tissue during the experimental period only. During this period, woody turnover mainly reflects branch shedding and a loss of heavily suppressed trees, and for this reason is likely to be longer than turnover times calculated over the whole lifetime of these species. Foliage and fine-root tissue have longer turnover times at Duke than at Oak Ridge. At both sites there was a noticeable CO_2_ effect on the lifespan of fine roots, although root lifespan decreased overall at Duke, whereas at Oak Ridge root lifespan increased at eCO_2_, likely because of deeper rooting distributions (Iversen *et al.*, 2008).

**Table 2:** Mean lifespan (years) of the foliage, fine roots and woody biomass at Duke

	Foliage	Fine roots	Wood (Ambient)	Wood (Elevated)
Observations	1.7	3.6	124.6	146.7
(1) Canopy foliar area optimisation				
CABLE	1.1	–*	66.1	66.6
CLM4	2.1	2.1	47.2	48.4
EALCO	1.5	18.8	143.0	124.3
GDAY	1.7	1.7	51.8	52.1
(2) Functional relationships				
ED2	2.3†	5.9	0.0†	0.0†
LPJ-GUESS	1.5	1.4	2092.2	2922.2
O-CN	1.4	1.5	268.8	254.4
(3) Resource limitations				
DAYCENT	1.8	5.0	207.7	200.9
ISAM	1.4	0.5	41.4	41.9
TECO	1.3	1.2	57.9	58.1
(4) Canopy foliar area optimisation				
SDGVM	2.8	10.1	55.6	77.0

Annual estimates of lifespan are calculated as the maximum of the biomass pool in a given year divided by the sum of the litter and mortality in that year; these estimates are then averaged over the years of simulation. Lifespans for woody biomass are given for Ambient and Elevated CO_2_ treatments.

* CABLE does not explicitly represent fine roots.

† ED2 assumed no mortality occurred during the course of the simulations at Duke. See Table 1 for details of the models.

**Table 3:** Mean lifespan (years) of the foliage, fine roots and woody biomass at Oak Ridge

	Foliage	Fine roots	Wood (Ambient)	Wood (Elevated)
Observations	0.6	0.9	203.1	218.7
(1) Canopy foliar area optimisation				
CABLE	1.1*	–†	64.4	64.8
CLM4	0.4	1.0	46.6	47.3
EALCO	0.4	18.9	239.4	224.9
GDAY	0.5	0.8	95.5	95.1
(2) Functional relationships				
ED2	0.3	3.7	175.0	178.5
LPJ-GUESS	0.3	1.3	10.8	9.5
O-CN	0.4	1.6	824.2	850.6
(3) Resource limitations				
DAYCENT	0.2	4.9	36.9	36.9
ISAM	0.4	1.1	43.0	43.8
TECO	0.3	2.0	61.4	62.3
(4) Canopy foliar area optimisation				
SDGVM	0.4	6.7	23.9	26.4

Annual estimates of lifespan are calculated as the maximum of the biomass pool in a given year divided by the sum of the litter and mortality in that year; these estimates are then averaged over the years of simulation. Lifespans for woody biomass are given for Ambient and Elevated CO_2_ treatments.

* CABLE has a foliage lifespan > 1 yr because it maintains a small leaf area index (LAI; *c*. 0.5–1) over winter from which it re-establishes a canopy when simulating deciduous plant functional types (PFTs). See Table 1 for details of the models.

† CABLE does not explicitly represent fine roots.

This suite of models poorly replicated the observed tissue lifespans during the experimental period (Tables2 and 3), with considerable variability across sites and among models, for all tissues. Many models tended to suggest shorter woody tissue lifespan than the observed, including CABLE, CLM4, GDAY, ISAM and SDGVM at Duke and CABLE, CLM4, DAYCENT, GDAY, ISAM, LPJ-GUESS, SDGVM and ISAM at Oak Ridge. A shorter lifespan is to be expected in models that set turnover rates based on the full lifetime of woody species. For example, GDAY and TECO have shorter woody lifespans than the observations because these models used general model parameterisations. Similarly, the DAYCENT model predicted woody turnover times that were six times longer at Duke compared with Oak Ridge. This was because at Duke, the death rate for woody biomass was set to zero and thus the plotted data only reflects litter from the branches.

By contrast, some of the models (ED2, LPJ-GUESS, O-CN and SDGVM) had a self-thinning mortality mechanism, which caused differences between sites and in response to eCO_2_ treatment (Tables3). However, these differences were not consistent among models. For example, in LPJ-GUESS woody litter is produced via either disturbance or mortality. For these simulations, stochastic disturbance and fire events were switched off, so woody litter was only produced by tree mortality, which increases as the canopy becomes denser and competition for light more severe. As the canopy was simulated to be less dense at Duke, partly because of lower SLA of the dominating conifers compared with the broad-leaved trees at Oak Ridge, less mortality occurred at Duke. By contrast, at Oak Ridge, mortality substantially decreased woody biomass. With respect to eCO_2_, SDGVM predicted an increase in woody turnover time. In SDGVM, self-thinning occurs when a diameter increment falls below a prescribed minimum. At eCO_2_, the increased productivity enables more trees to reach the minimum diameter increment, increasing woody lifespan.

### Consequences for carbon storage in biomass

**Fig 5:**
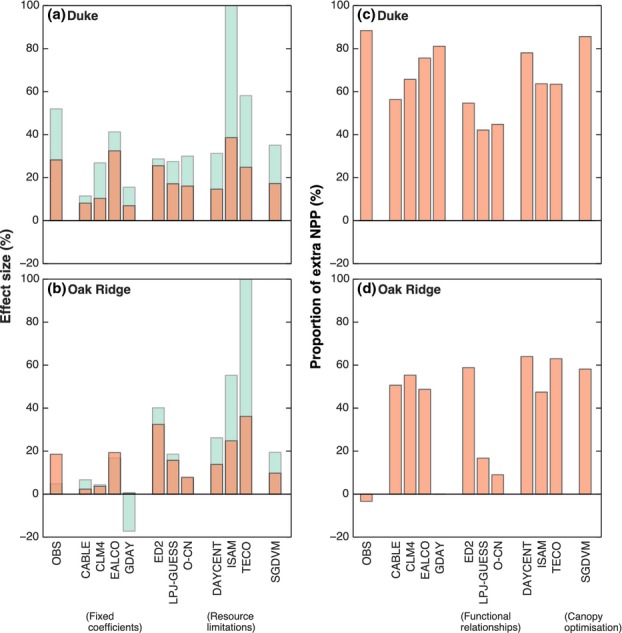
The effect of CO_2_ enhancement on vegetation carbon storage at the two sites. Left-hand plots show the effect of elevated CO_2_ on cumulative Net Primary Productivity (NPP; red bars) and biomass increment (blue bars) over the experiment at (a) Duke and (b) Oak Ridge. Right-hand plots show the proportion of additional NPP resulting from the increase in CO_2_ which remains in the plant biomass (foliage, wood and fine roots) at the end of the experiment at (c) Duke and (d) Oak Ridge. Note the bar for TECO in panel (b) has been clipped to 100% for plotting purposes, but extends to 109%. Observations are shown by the abbreviation ‘OBS’.

We compared the CO_2_ effect on NPP with the CO_2_ effect on biomass increment over the duration of the experiment (Fig. 5a,b). Most of the models predicted that the effect of eCO_2_ on biomass increment exceeded the effect of eCO_2_ on NPP. The difference between the CO_2_ effect on biomass increment and that on NPP depends on how far the simulated stand is from steady state, that is, the point where gains from NPP equal losses to turnover and mortality. In the very early stages of stand growth, before notable turnover or tree mortality commences, the simulated CO_2_ effect on biomass increment will be equal to the CO_2_ effect on NPP. At steady state, by contrast, the rate of biomass increment (at aCO_2_) is zero, so any stimulation of biomass increment by eCO_2_ will result in a very high relative response. This stand stage effect accounts for the large percentage increase in biomass seen in the ISAM model at both the Duke and ORNL FACE sites. A shift in allocation towards long-lived woody components will also increase the percentage biomass increment response compared to the NPP response, because woody tissue has a long lifespan. This effect can be seen in the TECO simulations, particularly at Oak Ridge where woody allocation increases by 10% (Fig. 3l), and as a result a 36% stimulation of NPP results in a 109% increase in biomass increment over the course of the experiment.

We also calculated the percentage of the increase in NPP due to eCO_2_ that was retained in biomass by the end of the experiment (Fig. 5c,d), which we called the NPP retention rate. Observations showed a dramatic difference between the Duke and ORNL FACE sites in the NPP retention rate (Fig. 5c,d). At Duke, 88% of the extra NPP due to eCO_2_ remained in biomass, whereas at ORNL, none of the additional NPP remained at the end of the experiment. This difference is remarkable given that the stimulation of NPP did not differ greatly between the experiments. This difference can be attributed to changes in allocation pattern: at Duke, there was a shift in the allocation of NPP to long-lived woody biomass, whereas at ORNL, the additional NPP was largely allocated to short-lived fine roots (Iversen *et al.*, 2008).

The predicted NPP retention rate varied strongly among the models. This percentage depends on the wood allocation fraction and wood turnover time, and was particularly sensitive to changes in either of these parameters with eCO_2_. At Duke, models suggested that a high proportion (i.e. > 40%) of the NPP enhancement remained in the tree biomass. The two models with low wood allocation at Duke (LPJ-GUESS, O-CN, Fig. 1) predicted the smallest NPP retention rate. SDGVM predicted the greatest NPP retention rate, despite a relatively low allocation to wood, because of the prediction that wood lifespan increases with eCO_2_ (Table 2). The TECO model also has a high NPP retention rate despite a low wood allocation at aCO_2_ (Fig. 1), because of the large increase in wood allocation fraction with eCO_2_ (Fig. 2).

At Oak Ridge, the models predicted a somewhat smaller NPP retention rate, largely as a result of lower wood allocation coefficients (Fig. 1), but few models captured the magnitude of the observed response. The GDAY model does capture the response, but this result was not predicted, but rather is a result of the prescribed change in allocation to roots based on the observations. LPJ-GUESS and O-CN predicted the smallest NPP retention rates. However, LPJ-GUESS makes this prediction not due to a shift in allocation towards roots, but rather because woody allocation is low (Fig. 1) and there is a very rapid woody turnover rate (Table 3). Similar to the observations, the low NPP retention rate in O-CN occurs because of a shift in allocation towards roots (Fig. 3) as well as the low wood allocation fraction.

## Discussion

Our goal in this paper was to address uncertainty in ecosystem models caused by different model assumptions about allocation and turnover processes. To do so, we applied 11 ecosystem models to data from two forest FACE experiments and used the experimental data to help discriminate among the model assumptions. These two forest FACE experiments provide uniquely rich datasets to constrain the response of allocation processes to CO_2_ in ecosystem models. Much of our previous understanding of allocation responses to eCO_2_ has come from meta-analysis using predominantly potted plants (Curtis & Wang, 1998; Poorter *et al.*, 2012). For example, Curtis & Wang (1998) found little evidence for sustained shifts in belowground allocation patterns due to CO_2_. Similarly, Poorter *et al.* (2012) found little evidence of a consistent CO_2_ effect on C allocation fractions (leaf, wood and roots) from a meta-analysis of young plants grown under controlled conditions. However, ecosystem models need to be informed by allocation patterns at ecosystem scale, rather than those in rapidly expanding young plants, where ontogenetic effects tend to outweigh environmental factors. Furthermore, to provide strong constraints on model behaviour, we need data on allocation patterns in response to experimental manipulations that are accompanied by detailed information on plant nutrient and water status. The intensively-studied FACE experiments are thus of tremendous value for evaluation of allocation models.

Nonetheless, it is important to recognise the limits to which these data can constrain models. First, there are significant uncertainties in the data due to the inherent difficulty of estimating biomass production in large forests. For example, estimates of woody biomass production were made using allometric equations determined from trees harvested before the onset of treatments. Root biomass production estimates were made by scaling measurements of root length measured using minirhizotron technology to root biomass (Iversen *et al.*, 2008; Pritchard *et al.*, 2008). Second, there were a number of one-off events that likely affected allocation patterns in the experiments, but were not related to atmospheric CO_2_ and are not captured in models. These events include a windstorm at Oak Ridge in 2004 and an ice storm at Duke in 2002 (McCarthy *et al.*, 2006). Third, changes in allocation patterns in the models are intended to represent responses to gradual changes rather than the step increase in CO_2_ concentration applied in the experiments. Furthermore, most models were parameterised with standard PFT parameters rather than site-specific parameters. Also, at the Duke site, the significant hardwood understorey is ignored by most models, which simulate pines only. For these reasons, we should not expect any model to precisely match the observed magnitude and interannual variability of treatment effects on allocation. Rather, we assessed the capacity of the models to qualitatively reproduce the major features of the observed changes. The overall effects of CO_2_ treatment on allocation patterns were clear, but differed between the two sites, with N availability as an important driver (Finzi *et al.*, 2007; Norby *et al.*, 2010; Zaehle *et al.*, 2014).

### Comparative success of different allocation models

We examined four different classes of allocation assumption. Broadly speaking, the models that used functional relationships among biomass fractions to control C allocation (ED2, LPJ-GUESS, O-CN) were best able to replicate the contrasting observed changes in C partitioning at eCO_2_ at both sites. These models initially predicted an increase in wood allocation with eCO_2_ in line with the observations, but as these models became water and nutrient stressed, allocation shifted towards roots. Thus, both the allometry of leaf to wood biomass, and the shift in the functional relationship between leaf and root biomass with stress, were important to capture the CO_2_ response. The timing of the development of stress responses varied between the models and differed from observations (see Zaehle *et al.*, 2014), but they did tend to capture the direction of allocation shifts due to eCO_2_. The success of these schemes is in contrast to previous work by Luo *et al.* (1994), who found that a model built on the principles of the functional balance hypothesis did a poor job of explaining observed changes in root allocation in response to eCO_2_. However, this study concerned young plants aged 22 d to 27 months. In addition, a key assumption of the model used by Luo *et al.* (1994) was that total N uptake did not change in response to CO_2_ treatment, as was observed in the experiments they considered. By contrast, N uptake increased at eCO_2_ in both of the FACE sites studied here (Finzi *et al.*, 2007). Thus, the functional balance approach appears to be more successful for explaining the CO_2_ effects on allocation in forest ecosystems than in young plants.

By comparison to the observations, modelled changes in allocation patterns were more gradual, meaning that they did not match the observed interannual variability in the observations. The models show a lagged response of allocation to changes in water and nutrient limitations (due to annual allocation in LPJ-GUESS, and a time-integrated N scalar in O-CN), which buffers the rate at which allocation to roots changes. However, as explained above, we would not necessarily expect the models to be able to simulate responses to step changes in environmental conditions. Of more concern is the fact that different parameterisations among these models resulted in marked differences among otherwise similar schemes (see Notes S2), indicating that parameterisation of these schemes is a source of significant uncertainty. Large-scale synthesis of data on allocation patterns (Litton *et al.*, 2007; Wolf *et al.*, 2011a,b) could potentially be used to reduce this uncertainty, particularly if synthesis was done in terms of model parameters.

The other three approaches used to represent allocation in our ecosystem models were considerably less successful at reproducing observations. Of particular concern, allocation schemes in which the allocation coefficients were not constrained by the resulting biomass fractions (i.e. constant coefficient and resource limitation approaches) could have unintended outcomes. For example, due to the interaction of allocation with a phenological scheme, CABLE unexpectedly predicted an eCO_2_ effect on C allocation to wood during a drought at Duke. Similarly, in ISAM, a maximum LAI was prescribed, causing leaf senescence in eCO_2_ to be delayed by as much as 20 d, with the unintended result of increased partitioning to foliage at eCO_2_ at ORNL. These results show that allocation schemes either need to be constrained by the resultant biomass fractions (e.g. the functional relationships approach) or tested thoroughly to ensure that model predictions are as intended.

The constant allocation coefficient approach (CABLE, CLM4, EALCO, GDAY) is unsuitable for predicting the consequences of eCO_2_ for allocation because it is unable to capture dynamic changes in allocation with changing water and nutrient availability at seasonal to interannual timescales. The experimental data show that these shifts in allocation pattern are significant, and therefore need to be captured in models, although it remains uncertain whether these changes in allocation pattern will be persistent over the long term.

The resource limitation approach – in which allocation fractions are decided based on the relative strength of nutrient, water, and light limitations – is similar in some ways to the functional relationships approach, but the models were significantly less successful at predicting the observed allocation patterns. This lack of success may be due to the fact that the approach is based on allocation fractions, which are considerably more difficult to measure than biomass fractions, with the consequence that many fewer data are available on which to base model formulations and parameters. In addition, at least some of the available data available do not support the general approach of prioritisation among plant components used in DAYCENT and TECO (Litton *et al.*, 2007).

The one optimisation approach to allocation included in our set of 11 models (SDGM) also failed to capture the observed responses. However, this was principally because the optimisation approach was incomplete, combining foliar optimisation with fixed coefficients for wood and root tissues. A number of other optimisation and game-theoretic allocation models have been developed (e.g. see Franklin *et al.*, 2012). Several of these approaches have given promising results for explaining observed patterns in C allocation (Dewar *et al.*, 2009; Dybzinski *et al.*, 2011; Valentine & Mäkelä, 2012; McMurtrie & Dewar, 2013) including observations from FACE experiments (Franklin *et al.*, 2009; McMurtrie *et al.*, 2012). The results from these studies are sufficiently promising to merit investigation of the implications of these concepts when implemented into ecosystem models. It would be particularly useful to implement the ‘assumption-centred’ model evaluation framework developed here to investigate how such models compare to the allocation models currently in use.

### Other important processes

In addition to allocation, tissue turnover is a key process determining C storage in biomass, particularly turnover rates of the long-lived woody biomass (Bugmann & Bigler, 2011; Smith *et al.*, 2013; Xia *et al.*, 2013). Very few of the models considered here include any explicit mechanism governing turnover. Tissue lifespan is usually a prescribed parameter, either by PFT or based on site knowledge. Elevated CO_2_ has been shown to affect tissue lifespan. For example, needle lifespan was reduced at Duke FACE (Schäfer *et al.*, 2002) and root lifespan was increased at ORNL FACE (Iversen *et al.*, 2008). This CO_2_-induced response has implications for short-term litterfall and long-term soil C storage (see Iversen *et al.*, 2012). Even the models that employed a mechanism to adjust lifespan still did not compare well to data: LPJ-GUESS and SDGVM produced very different and at times unrealistic results when applied to a transient step-change experiment. Amongst models in which turnover processes are parameterised, there was striking inter-model variability in the lifespan of the wood, foliage and fine roots (Tables3); it varies by as much as an order of magnitude for the woody component. These results point to a need for better data on turnover. Such data could come from many sources besides manipulative CO_2_ experiments. In particular, they need to cover all stages in forest development (Wolf *et al.*, 2011b).

Similarly, to estimate CO_2_ effects on canopy cover, models need to estimate SLA in addition to foliage allocation. Most models prescribed SLA and therefore did not capture the observed reduction in SLA due to eCO_2_. As a consequence, changes in canopy cover in response to eCO_2_ are overestimated. However, the only model currently incorporating a theoretical prediction of SLA (CLM-CN) performed worse, because SLA was predicted to increase rather than decrease. A reduction in SLA is a commonly observed response in eCO_2_ experiments (Medlyn *et al.*, 1999; Ainsworth & Long, 2005; Poorter *et al.*, 2009) that needs to be incorporated in ecosystem models, preferably via a process-based prediction of SLA rather than an *ad hoc* reduction in SLA as CO_2_ increases. SLA is one of the most commonly studied plant traits (Kattge *et al.*, 2011), so there are ample data available on which to base such a model.

### Where does the carbon go?

The observed site responses show contrasting effects of eCO_2_ on the fate of vegetation C. There was a sustained increase in biomass C at Duke FACE but no sustained increase at ORNL FACE. In both cases, models were unable to correctly simulate the change in C storage, because they were unable to capture the full extent of the site N dynamics (see Zaehle *et al.*, 2014) and the resulting change in allocation patterns. At Duke FACE, models tended to predict that a greater proportion of the enhancement in NPP remained in the plant biomass at the end of the experiment than the observations indicated. In many cases (DAYCENT, EALCO, ED2, LPJ-GUESS and O-CN), this was because the models prescribed too long a turnover time for wood, and allocated too much of the additional NPP to wood. The response was more variable at Oak Ridge, but models again over-predicted the resulting change in plant biomass (with the exception of GDAY, which used prescribed allocation). At both sites, therefore, models generally over-predicted the C storage due to eCO_2_.

Soil is also a major store for carbon. We did not address the CO_2_ effect on C storage in the soil, as we were focusing on model assumptions related to biomass allocation and turnover. Predictions of soil C storage will be influenced by the input of C to soil, which is dependent on assumptions about allocation, especially to fine roots (Iversen *et al.* 2012), but the fate of C in soil depends on a different set of model assumptions that are chiefly related to organic matter decomposition. Future work should investigate how these assumptions differ among models and the interaction between plant allocation and soil processes. Constraining these assumptions with data will be challenging, given the inherent uncertainty in soil C data (Hungate *et al.*, 2009). Even after a decade of experimentation, soil C changes in the two FACE experiments are difficult to detect because of the large, heterogeneous background pool.

We also do not address the allocation of photosynthate to processes other than growth and respiration. These processes include C exudation to the rhizosphere, transfer to mycorrhizae, volatile organic C emissions, and losses to herbivory. These C flows may have important ecosystem consequences; for example, rhizosphere C inputs are thought to increase with eCO_2_, stimulating microbial activity and enhancing plant available N (Drake *et al.*, 2011; Phillips *et al.*, 2012). However, these fluxes have not been quantified directly for the two FACE sites, and estimates have principally been inferred from mass balance calculations (Palmroth *et al.*, 2006; Drake *et al.*, 2011; Phillips *et al.*, 2011). Furthermore, none of the models considered here have any mechanistic representation of rhizodeposition processes. Consequently, these additional C flows remain a key unknown requiring additional experimental data and model development.

Some of the models (ED2, LPJ-GUESS and O-CN) did include allocation of C to reproduction. Where these fluxes were simulated, they were considerably larger than observed. In the case of ED2, for example, the allocation fraction to reproduction was 16–22% and increased by 6–12% with eCO_2_. By contrast, the observed allocation to reproduction was <1% at Duke (McCarthy *et al.*, 2010). The sweetgum trees at ORNL did not produce measurable reproductive tissue within the timeframe of the experiment.

### Ways to reduce model uncertainty

This study has shown that model uncertainty due to allocation and turnover processes could be reduced through several means, including improvements to models, targeted synthesis of experimental data and additional measurements.

We have shown that allocation approaches that are constrained by biomass fractions (such as functional relationships) were more successful at capturing observed trends, and were generally more robust, than approaches based on allocation coefficients. In particular, we showed that approaches using constant allocation coefficients or resource limitations, when combined with phenological schemes occasionally produced unintended responses to eCO_2_. We therefore advocate allocation approaches based on functional relationships or optimisation schemes, and that any allocation model should be subjected to wide-ranging tests to discover whether it behaves as intended.

We have shown that allocation parameters differ considerably among models. Synthesis of existing allocation data, especially if it is done in terms of model parameters, would reduce uncertainty among models by providing baseline parameter values. Similarly, we showed that turnover coefficients were highly variable among models, indicating that they are poorly constrained by data. Uncertainty among models could be reduced with better measurements of turnover, as well as synthesis of existing measurements. Such work could also assist in developing better models of turnover. SLA has been extensively measured, and these measurements should be used to help develop process representation for environmental effects on SLA.

FACE experiments provide rich datasets with which to constrain models, but the strongly contrasting responses between the two experimental sites imply that additional datasets will be needed to derive generalisations about allocation at the ecosystem scale. Ecosystem manipulation experiments need to be intensively studied to provide all the data needed to constrain models. For the work presented here we required data on growth and turnover of all plant components as well as complementary data on plant water and nutrient availability. We recommend that future ecosystem-scale experiments attempt to fully quantify carbon, water and nutrient budgets.

## Supporting Information

Fig S1 Modelled labile carbohydrate store at Duke and at Oak Ridge. **Notes S1** Carbohydrate storage and remobilisation. **Notes S2**Differences among model predictions of allocation patterns at ambient CO_2_ concentration.
