# Supplementary material for: Where does the carbon go? A model–data intercomparison of vegetation carbon allocation and turnover processes at two temperate forest free-air CO2 enrichment sites
Source: New Phytol. 2014 May 21;203(3):883–99. doi: 10.1111/nph.12847 (PMC4260117; doi:10.1111/nph.12847)
Supplement: Fig S1 — Modelled labile carbohydrate store at Duke and at Oak Ridge. Notes S1 Carbohydrate storage and remobilisation. Notes S2Differences among model predictions of allocation patterns at ambient CO2 concentration. [file nph0203-0883-SD1.doc]

**Supporting Information Notes S1 & S2 and Fig. S1(a, b)**

**Notes S1 Carbohydrate storage and remobilisation**

In some models (e.g. CABLE, ISAM, CLM4 and GDAY applied to evergreen species), all carbon taken up through photosynthesis is used for respiration and growth on the same day. In other models, however, the timing of photosynthesis and growth is assumed to differ, so carbon taken up through photosynthesis is stored in a labile carbon pool to be used for growth and respiration at a later date. The timing of labile carbon use depends on the phenology scheme applied. The key difference among models using this approach lies in how they determine the amount of carbon to be taken out of this pool and used for growth.

In several models (e.g. ED, GDAY, TECO, LPJ-GUESS), the extraction of carbon from the labile carbon pool depends on the amount placed in it during the previous photosynthesis period. In these models, the labile C is returned to zero at a given time point each year (Fig. S1a and b). These models therefore do not allow a build-up of labile carbon over time.

In other models (e.g. CLM4 for deciduous species, O-CN, SDGVM) the extraction of carbon from the labile C pool depends on the pool size. For example, for deciduous species in CLM4, total plant growth each year is equal to half of the labile carbon pool. Since

dC_labile_ / dt = NPP - Growth = NPP – 0.5 C_labile_ ,

this implies that in equilibrium, the labile C pool must be equal to twice the size of annual average NPP. In these models, therefore, the labile C pool can be quite large (Fig. S1a and b). It also increases in size with increasing CO_2_, in proportion to the increase in NPP.

Carbohydrate storage is represented in most detail in O-CN. In this model, the non-respiring labile carbon reserve is used to speed-up foliar development during the leaf-out phase, therefore the target size for this pool is the sum of the deciduous plant organs (leaves and fine roots) of the previous year. The pool is replenished during the remainder of the year, whenever current GPP exceeds the target size of the meristem pool. Nutrient limitation may prevent the full usage of the carbon, such that it is also possible for labile carbon to build up over time when nutrient uptake is insufficient to support demand for growth. Once the labile C pool reaches a threshold size, respiration rates increase to remove the excess labile C.

The assumptions in the models are evidently simplistic. The different assumptions reflect different approaches to carbon accounting within models, rather than different physiological hypotheses about the mechanisms involved in labile carbon storage and re-use. The models have been developed largely in the absence of experimental data on rates of labile C storage and depletion, and how these rates vary with photosynthetic uptake, species phenology and environmental stress. New information is becoming available that may improve these assumptions (e.g. Gaudinski *et al*., 2009; Gough *et al*., 2009, 2010; Richardson *et al*., 2012) and this will be a key area for data synthesis and model improvement in the near future.

**Fig. S1(a)** Modelled Labile Carbohydrate store at Duke. Empty panels indicate that the model does not simulate a labile carbohydrate storage pool.

**Fig. S1(b)** Modelled Labile Carbohydrate store at Oak Ridge. Empty panels indicate that the model does not simulate a labile carbohydrate storage pool.

**Notes S2 Differences among model predictions of allocation patterns at ambient CO_2_ concentration**

1. *Fixed coefficients*

Both CLM4 and GDAY specified constant allocation coefficients based on the measured site data and thus replicate the observed allocation patterns at ambient [CO_2_]. CLM4 does not exactly match the site data as these coefficients are based on a smaller sample of the site data. The other two fixed-coefficient models, CABLE and EALCO, used generic allocation coefficients according to PFT, explaining the differences among the fixed-coefficient models at ambient CO_2_ (Fig. 1).

1. *Functional relationships*

The three models ED2, LPJ-GUESS and O-CN allocate C according to functional relationships among plant organs, parameterised according to PFTs. Differences in allocation patterns at aCO_2_ among these models result from differing parameterisations. Both LPJ-GUESS and O-CN had similar foliage and root allocation at Duke and ORNL, whereas ED2 had much greater foliage than root allocation at Duke than at ORNL (Fig. 1). For LPJ-GUESS and O-CN, the baseline root-to-foliage ratio is the same for both PFTs. For ED2, this ratio is initialised at a lesser value for coniferous PFTs than for broadleaf PFTs, explaining why the foliage allocation is so much greater than root allocation for ED2 at Duke. LPJ-GUESS shows marked differences in allocation patterns between the sites, with much greater wood allocation at Duke than at Oak Ridge which results from growing taller stands at Duke (Fig. 1). This difference is not seen in the O-CN simulations. The models differ because O-CN assumes that the C costs for the support structures of leaves, as described by the ratio of leaf area to sapwood area, does not vary between PFTs, whereas LPJ-GUESS assumes a lesser cost at Oak Ridge than Duke. As a result, LPJ-GUESS predicts greater allocation to wood, without the requirement of a corresponding allocation to the foliage and fine roots, at Oak Ridge.

1. *Resource limitations*

The ISAM model predicts considerably lower root allocation at Duke than at Oak Ridge (Fig. 1), largely because allocation is assumed to differ among phenological stages at Oak Ridge. At Duke, the largest allocation fraction is to wood, because LAI is sufficiently high that light is limiting. Root allocation is low throughout the year because of the lack of water limitation at the site. A similar pattern occurs at Oak Ridge during the main growing season, but this phase only lasts about 70 days, and the early and late phenological phases change the allocation proportions. In the early phase, all the NPP is allocated to grow leaves and no growth of the other components takes place. During the late phase (leaf senescence), there is no foliage allocation, wood allocation decreases as there is no light limitation, and instead root allocation increases. The result of this phenological phasing is to increase root allocation at Oak Ridge relative to Duke.

The DAYCENT model prioritises the allocation of NPP firstly to fine roots, then to foliage and then, if there is still C available, to the woody tissue. At aCO_2_, allocation to foliage is slightly greater, and allocation to roots is slightly lower, at Duke than at Oak Ridge, due to differences in parameterisation between the sites.

The TECO model also uses a prioritisation scheme, but the differences between sites predicted by this model are much larger and in the opposite direction from DAYCENT, with much greater foliage allocation and lesser root allocation at Oak Ridge. At Duke, the allocation coefficients are close to their prescribed maxima (30% of NPP for roots and 40% of NPP for foliage). At Oak Ridge, the annual average foliage allocation is above the prescribed daily maximum (40% of NPP) because, for deciduous canopies, TECO also allocates early season growth from a non-structural carbohydrate store to facilitate the initial growth of leaves.

1. *Optimisation*

The LAI optimisation scheme used in the SDGVM model results in a greater foliage allocation at Oak Ridge than Duke (Fig. 1). This prediction is a consequence of the shorter leaf lifespan at Oak Ridge. The optimal LAI was not greatly different between the two sites, so greater foliage allocation is required to compensate for the shorter leaf life span. The root and wood allocation fractions are fixed fractions of the labile C remaining after foliage allocation, so allocation to root and wood are both lesser at Oak Ridge compared to Duke.

**References**

**Gaudinski JB, Torn MS, Riley WJ, Swanston C, Trumbore SE, Joslin JD, Majdi H, Dawson TE, Hanson PJ. 2009.** Use of stored carbon reserves in growth of temperate tree roots and leaf buds: analyses using radiocarbon measurements and modeling. *Global Change Biology* **15**: 992–1014.

**Gough CM, Flower CE, Vogel CS, Dragoni D, Curtis PS. 2009.** Whole-ecosystem labile carbon production in a north temperate deciduous forest. *Agricultural and Forest Meteorology* **149**: 1531–1540.

**Gough CM, Flower CE, Vogel CS, Dragoni D, Curtis PS. 2010.** Phenological and temperature controls on the temporal non-structural carbohydrate dynamics of *Populus grandidentata* and *Quercus rubra*. *Forests* **1**: 65–81.

**Richardson AD, Carbone MS, Keenan TF, Czimczik CI, Hollinger DY, Murakami P, Schaberg PG, Xu X. 2013.** Seasonal dynamics and age of stemwood nonstructural carbohydrates in temperate forest trees. *New Phytologist* **197**: 850–861.
